# Strategy for Accurate Detection of Six Tropane Alkaloids in Honey Using Lateral Flow Immunosensors

**DOI:** 10.3390/s24227265

**Published:** 2024-11-13

**Authors:** Boyan Sun, Chuanlei Wang, Zile Wang, Jiayi Liang, Ke Han, Shuai Zhang, Chunchao Yin, Xiaomei Wang, Chujun Liu, Zhiyue Feng, Sihan Wang, Haiyang Jiang

**Affiliations:** 1 National Key Laboratory of Veterinary Public Health Security, Beijing Key Laboratory of Detection Technology for Animal-Derived Food Safety, Beijing Laboratory for Food Quality and Safety, Department of Veterinary Pharmacology and Toxicology, College of Veterinary Medicine, China Agricultural University, Beijing 100193, China; boyan5758@163.com (B.S.); chuanlei1113@163.com (C.W.); wangzile2017@163.com (Z.W.); hankeee@126.com (K.H.); zsxxsc@163.com (S.Z.); yinchunchao1222@163.com (C.Y.); xiaomeiwangg@163.com (X.W.); liuchujuncjl@163.com (C.L.); f_zy99@163.com (Z.F.); 2 Chinese Academy of Inspection and Quarantine, Beijing 100176, China; 3 Department of Chemistry, Waterloo Institute for Nanotechnology, University of Waterloo, Waterloo, ON N2L 3G1, Canada; j284lian@uwaterloo.ca

**Keywords:** AuNP-LFIA, tropane alkaloids, honey, multi-target, lateral flow immunoassay

## Abstract

Honey, a widely consumed food, is susceptible to contamination by various toxic substances during production. Tropane alkaloids, with their potent neurotoxicity, are frequently found in honey. Hence, there is an acute need for rapid and effective detection methods to monitor these alkaloids. Lateral flow immunoassay (LFIA), known for its simple operation, low cost, and reliable results, holds great promise. In this study, we developed an efficient and user-friendly analytical method for the simultaneous detection of six tropane alkaloids (atropine, L-hyoscyamine, scopolamine, anisodamine, homatropine, and apoatropine) in honey based on an AuNPs lateral flow immunoassay (AuNPs-LFIA) with broad-spectrum antibodies. Under optimal conditions, the calculated detection limits were 0.22, 0.29, 0.51, 6.34, 0.30, and 0.94 ng/mL, respectively. By diluting the honey sample five times, the contaminants can be readily detected using LFIA. Semi-quantitative and quantitative analyses can be completed within 17 min. This innovative method fills the void in LFIA for detecting tropane alkaloids and serves as a valuable reference for LFIA detection of honey samples, providing a crucial strategy for the accurate detection of these important compounds.

## 1. Introduction

Tropane alkaloids (TAs) are a class of compounds characterized by an octane skeleton with a nitrogen bridge, primarily found in plants of the Solanaceae family, including the genera Atropa and Datura [1,2]. TAs are major anticholinergic drugs that affect both the central and autonomic nervous systems, exhibiting strong neurotoxic effects [3]. When administered in excessive doses, TAs can cause severe toxicity, with common symptoms of poisoning including dizziness, pupil dilation, dry mouth, vomiting, confusion, muscle spasms, tachycardia, and in severe cases, death [4,5,6]. TAs in plants can enter honey through bees collecting nectar. After contaminated honey flows into the market, it poses a potential threat to human health [7]. Therefore, monitoring TA residues in animal-derived foods is a crucial task for ensuring human health. The European Food Safety Authority (EFSA) has conducted a risk assessment of TAs in food and feed, establishing an acute reference dose of 0.016 μg/kg for atropine (ATR) and scopolamine (SCO) and recommending scientific attention to the contamination levels of ATR and SCO in plant-based foods such as cereals. In 2015, the European Commission stipulated that the levels of ATR and SCO in agricultural products should not exceed 10 μg/kg, preferably below 5 μg/kg, and in ingredients, food supplements, herbal teas, and processed foods, the levels should be below 2 μg/kg [8]. In 2016, the EU established a legal limit of 1 μg/kg for ATR and SCO in infant food (EU 2016/239) [9].

Various methods for detecting TAs have been reported, including capillary electrophoresis (CE) [10], liquid chromatography-tandem mass spectrometry (LC-MS/MS) [11], high-performance liquid chromatography (HPLC), and ultra-high-performance liquid chromatography (UHPLC) [12,13,14,15]. These instrumental methods rely on expensive equipment, are difficult to operate, and require well-trained personnel, making them unsuitable for rapid on-site detection. Two of the immunological analysis methods based on the specific binding reaction of antibodies and antigens are enzyme-linked immunosorbent assay (ELISA) and lateral flow immunoassay (LFIA) [16]. ELISA involves complex detection procedures and longer incubation times, making it a laboratory-based platform not suitable for on-site detection [17,18]. In contrast, LFIA, being based on AuNPs, has the advantages of simplicity, rapidity, and low cost. In recent years, it has been widely used in food safety, environmental monitoring, and medical diagnosis [19]. However, an LFIA method for detecting TAs has not been reported yet.

**Figure 1:**
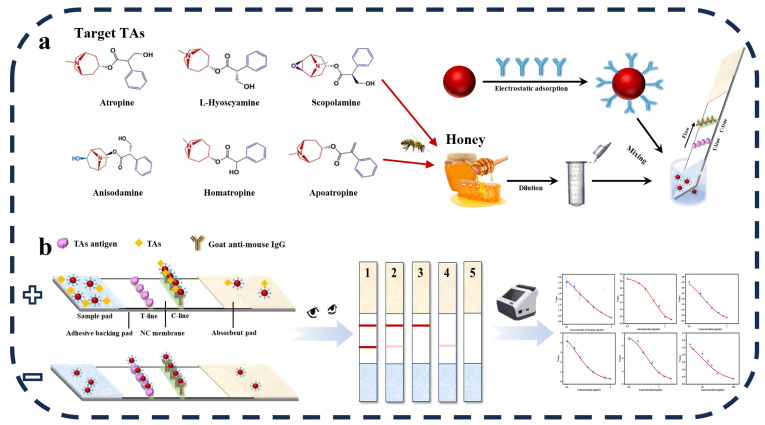
Schematic illustration of AuNPs-LFIA for detecting six tropane alkaloids (TAs). (**a**) Schematic workflow of the detection of 6 TAs in honey. (**b**) Semi-quantitative and quantitative signal reading methods and AuNPs-LFIA test strip coloration results: 1, negative; 2, weak positive result; 3, positive result; 4-5, invalid results.

In this study, we focused on the accurate detection of six tropane alkaloids (TAs) in honey. Leveraging a broad-spectrum TAs monoclonal antibody [9], we established an AuNPs lateral flow immunoassay (AuNPs-LFIA) specifically designed for detecting multiple TAs in honey. This innovative test strip has the remarkable ability to simultaneously analyze the content of atropine, L-hyoscyamine, scopolamine, anisodamine, homatropine, and apoatropine. It exhibits excellent sensitivity toward all six TAs, as illustrated in Figure 1. The results of spiked recovery tests in honey clearly demonstrated the outstanding practicality and stability of this LFIA method. Our research presents a simple, economical, and rapid LFIA approach for monitoring TA residue levels, effectively filling the gap in broad-spectrum LFIA methods for TA detection. This not only provides a crucial strategy for accurate detection but also offers a new technical means for safety inspections of animal-derived foods, particularly honey.

## 2. Materials and Methods

### 2.1. Reagents and Instruments

Atropine (ATR), L-hyoscyamine (LHY), scopolamine (SCO), anisodamine (ANI), homatropine (HOM), and apoatropine (APO) were purchased from Dr. Ehrenstorfer (Augsburg, Germany), Toronto Research Chemicals (Toronto, Canada), BOC Sciences (Shanghai, China), Tokyo Chemical Industry Co., Ltd. (Tokyo, Japan), Beijing Coupling Technology Co., Ltd. (Beijing, China) and MedChem Express (Shanghai, China), respectively. Additional compounds such as senecionine, seneciphylline, retrorsine, lucosamine, sanguinarine, macrotomine, aconitine, gelsemine, methylatropine, valatropine, methylhomatropine bromide, norhyoscine, clidinium bromide, rosmarinine, hyoscyamine, and allohyoscyamine were obtained from Yuan Ye Biotechnology Co., Ltd. (Shanghai, China). Bovine serum albumin (BSA), polyvinylpyrrolidone (PVP), Tween 20, and Triton X-100 were purchased from Sigma-Aldrich (St. Louis, Missouri, USA). Sucrose, HAuCl_4_•4H_2_O, trisodium citrate, K_2_CO_3_, PEG 20000, trehalose, NaCl, NaH_2_PO_4_·2H_2_O, Na_2_HPO_4_·12H_2_O, KCl, and methanol were procured from China National Pharmaceutical Group (Shanghai, China). Goat anti-mouse immunoglobulin (IgG), PVC backing cards, sample pads, nitrocellulose membranes, and absorbent pads were provided by Beijing WDWK Biotechnology Co., Ltd. (Beijing, China). The monoclonal antibody TRO 2B10 and the complete antigen TRO-EDC-BSA were produced in our laboratory. Commercial honey and beverages were all purchased from local supermarkets.

Zeta potential and hydrodynamic diameter measurements were performed using a ZetaSizer Nano ZS90 from Malvern Instruments (Malvern, UK). High-resolution transmission electron microscopy (HRTEM) analysis was conducted using a Tecnai G2 F20 S-TWIN field emission HRTEM from FEI Company (Hillsboro, Oregon, USA). The assembly of test strips was performed using an XYZ 3060 dispenser and ZQ2000 guillotine cutter from Shanghai Kinbio Tech Co., Ltd. Test strip data were read using an ESEQuant LR3 from QIAGEN (Düsseldorf, Germany). Photographs were taken with a Xiaomi 12s smartphone from Xiaomi Corporation (Beijing, China). Purified water was obtained using a Milli-Q ultrapure water system.

### 2.2. Preparation of AuNPs

Au nanoparticles (AuNPs) were prepared using the classical citrate reduction method [20]. Briefly, 20 mg of HAuCl_4_•4H_2_O was added to 50 mL of deionized water and heated in a water bath at 100 °C for 20 min. Under vigorous stirring and continued heating, 2.5 mL of 10 mg/mL trisodium citrate solution was rapidly added to the beaker. The reaction continued until the solution changed from pale yellow to a uniform wine-red color, indicating the formation of AuNPs. The prepared AuNPs were cooled to room temperature and stored at 4 °C for future use.

### 2.3. Preparation of AuNPs Immunoprobes

The AuNPs were conjugated with TA monoclonal antibodies via electrostatic adsorption. To 1 mL of AuNPs, 10 μL of 0.1 M potassium carbonate was added and mixed thoroughly, followed by the addition of 15 μL of 1.5 μg/mL TA monoclonal antibody. The mixture was allowed to react for 10 min. Subsequently, 100 μL of 10% BSA solution was added to block any excess binding sites on the AuNPs surface, and the reaction proceeded for another 10 min. The mixture was centrifuged at 11,963 rcf for 10 min, the supernatant was discarded, 200 μL of PBS containing 0.1% BSA and 0.005% PEG 20,000 were added to redissolve, then after ultrasonic dispersion, the resulting solution was stored at 4 °C.

### 2.4. Preparation of Immunochromatographic Test Strips

The components of the test strips include NC membranes coated with capture reagents, absorbent pads, sample pads, and PVC backing cards. Using a dispenser, TRO-EDC-BSA (1.8 mg/mL) and goat anti-mouse secondary antibody (1.4 mg/mL) were dispensed onto the NC membrane, forming the control line (C-line) and test line (T-line), respectively. The membranes were then dried at 37 °C for 0.5 h. All components were sequentially assembled onto PVC backing cards with an overlap of approximately 1–2 mm between adjacent components. Finally, the assembled strips were cut into 3 mm wide strips and stored at 4 °C until use.

### 2.5. Sample Preparation

A 5.0 g honey sample was placed into a 50 mL centrifuge tube. Then, 25 mL of PBS (pH = 7.58) + 1% Tween 20 solution was added and vortexed thoroughly to disperse the honey uniformly.

### 2.6. Detection Procedure

The test strips and AuNPs immunoprobes stored at 4 °C were placed at room temperature to warm up before use. One hundred μL of the sample solution was added to a well in a microplate, followed by 6 μL of the AuNPs immunoprobe. The mixture was thoroughly mixed. After a 3-min incubation, one end of the test strip’s sample pad was vertically immersed into the corresponding well for 14 min of chromatography. The semi-quantitative results were visually determined by the naked eye, with the concentration of TAs at which the T-line disappeared used as the visual limit of detection (vLOD). The test strip was then placed into a strip reader to read the T/C value. Taking the concentration of TAs as the abscissa and the value of T/C as the ordinate, we fitted the standard curve using Origin 2024 software, and took IC10 in the four-parameter fitting as the calculated detection limit (cLOD) [9]. The reading results of positive samples were input into the standard curve to achieve quantitative analyses of TA concentration in the samples.

### 2.7. Method Evaluation

By performing quantitative analysis on a series of standard solutions with known concentrations, standard curves, cLOD, detection linear range, and inhibitory parameters for the six TAs were obtained. The cross-reactivity rates of the six TAs were determined by comparing the IC50 values of the other five TAs to that of ATR. High, medium, and low concentrations of the six TAs were spiked into blank honey samples, which were then diluted with sample diluent and tested. The T/C values were read and input into the honey matrix calibration curve to obtain the average recovery rate and coefficient of variation (CV%). The practicality and accuracy of the method were evaluated based on the average recovery rate and CV% of the spiked samples. The specificity of the method was determined by reading the detection results for the six target TAs (100 ng/mL) and eight other alkaloids (1000 ng/mL).

## 3. Results and Discussion

### 3.1. Characterization of AuNPs and AuNPs-Abs

**Figure 2:**
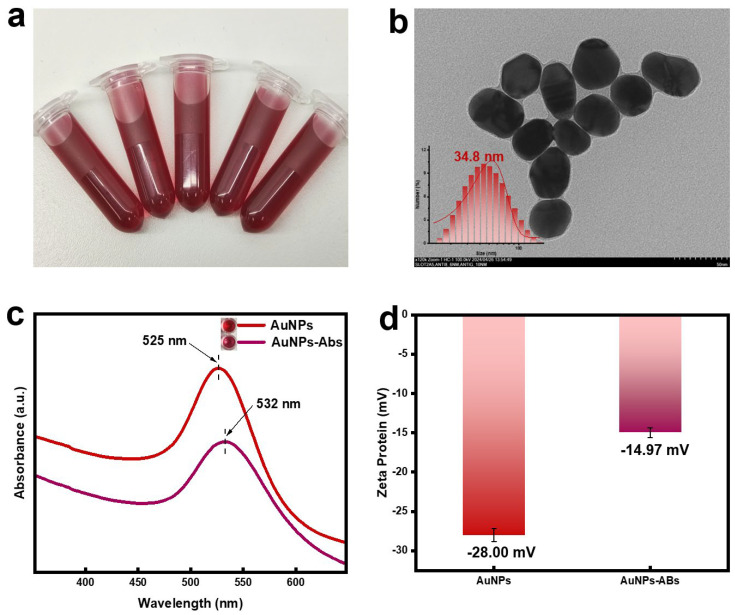
Characterization of AuNPs and AuNPs−Abs. (**a**) AuNPs. (**b**) HRTEM and DLS results of AuNPs. (**c**) UV−Vis absorption spectra of AuNPs and AuNPs−Abs. (**d**) Zeta potential of AuNPs and AuNPs−Abs.

As shown in Figure 2a, the synthesized AuNPs exhibited a wine-red color under natural light, appearing clear and bright without aggregation or precipitation. The HRTEM results (Figure 2b) indicated that the AuNPs were spherical or ellipsoidal in shape with a particle size of approximately 30 nm and good dispersion, consistent with the DLS results. The color of the AuNPs immunoprobe (AuNPs-Abs) prepared by electrostatic adsorption of antibodies was darker than the AuNPs (Figure 2c). The UV-Vis scanning spectrum results showed that the characteristic absorption peak of the AuNPs immunoprobe was red-shifted compared to that of the AuNPs. As shown in Figure 2d, the Zeta potential intensity of the immunoprobe formed by coupling TA antibodies with strongly negatively charged AuNPs was lower than that of the AuNPs. These results indicate that the TA monoclonal antibodies were successfully conjugated onto the AuNPs.

### 3.2. Optimization of Conditions

#### 3.2.1. pH Value

**Figure 3:**
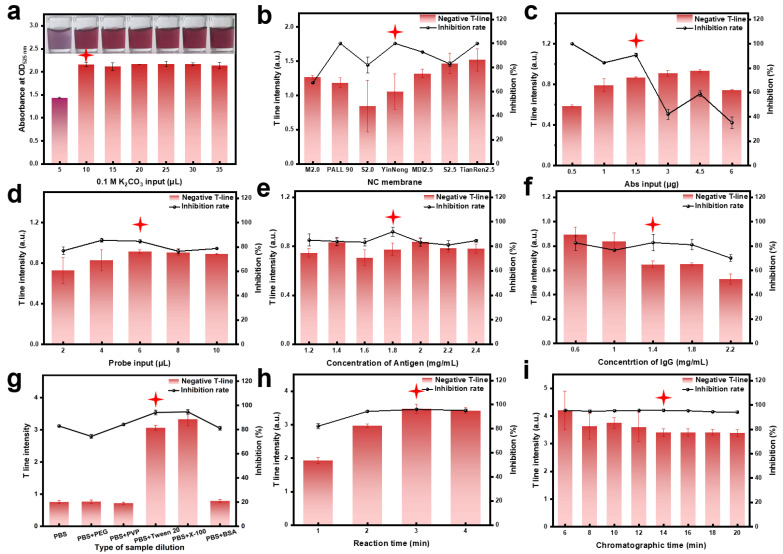
Optimization results of detection conditions. (**a**) Optimization of K_2_CO_3_ input amount. (**b**) Optimization of NC membrane type. (**c**) Optimization of TAs antibody input amount. (**d**) Optimization of AuNPs-Abs input amount. (**e**) Optimization of TRO-EDC-BSA concentration. (**f**) Optimization of IgG concentration. (**g**) Optimization of sample diluent. (**h**) Optimization of incubation time. (**i**) Optimization of chromatography time.

An appropriate pH is crucial for preparing AuNPs immunoprobes. Theoretically, the reaction pH should be slightly higher than the isoelectric point (pI) of the protein. Below the pI, antibodies tend to aggregate, leading to the precipitation of AuNPs-Abs and reduced detection accuracy. Above the pI, electrostatic repulsion between the antibodies and AuNPs limits adsorption, resulting in weaker color development on the test strips [21]. Therefore, the reaction system needs to be adjusted to an optimal pH that prevents AuNPs-Abs precipitation while ensuring sufficient color intensity. In this study, the solution pH was adjusted using 0.1 M K_2_CO_3_. As shown in Figure 3a, when the amount of K_2_CO_3_ was greater than or equal to 10 μL, no significant precipitation of AuNPs-Abs was observed. Considering the color intensity on the test strips, 10 μL of K_2_CO_3_ was selected as the optimal amount.

#### 3.2.2. NC Membrane

To achieve the desired signal intensity and optimal sensitivity, other conditions were optimized based on the color intensity of the T-line in the negative control group and the inhibition rate of the test strips with 10 ng/mL ATR.

The NC membrane is a key component of LFIA, responsible for immobilizing the TRO-EDC-BSA and IgG to form the T-line and C-line, respectively, and guiding the flow of the sample and detection reagents to the reaction zone. Different NC membranes have varying physical and chemical properties (such as porosity, thickness, and wettability), which affect chromatography and color development [22]. As shown in Figure 3b and Figure S1a, the “Yin neng” NC membrane provided good color intensity for the T-line in the negative control and high inhibition rates in the positive samples. Therefore, the “Yin neng” NC membrane was selected for further optimization.

#### 3.2.3. Sample Pad

In LFIA, the sample pad acts as a preliminary filter to remove large particles and impurities from the sample, ensuring detection accuracy and reliability [23]. As shown in Figure S1b, the sample solution exhibited good chromatographic performance on the SB08 glass fiber, whereas it failed to migrate to the NC membrane on the blood filter membrane to complete the corresponding reaction. Thus, SB08 glass fiber was chosen as the sample pad for further studies.

#### 3.2.4. Optimization of the Input Amount of the TA Monoclonal Antibody

As shown in Figure 3c and Figure S1c, when the antibody concentration was less than 4.5 μg/mL, the color intensity of the T-line in the negative control increased with the antibody concentration. However, once the antibody concentration went beyond 4.5 μg/mL, the color intensity of the T-line in the negative control notably diminished. When the antibody concentration was greater than 3.0 μg/mL, the inhibition rate markedly declined. Considering the color intensity of the T-line in the negative control and the inhibition rate, an antibody concentration of 1.5 μg/mL was selected for subsequent studies due to its strong color development and high inhibition rate.

#### 3.2.5. Amount of AuNPs-Abs

The optimization results for the amount of AuNPs-Abs are presented in Figure 3d and Figure S1d. With the increase in the amount of AuNPs-Abs, the color intensity of the T-line in the negative control gradually rose and stabilized at 6 μL. Once the amount of AuNPs-Abs exceeded 6 μL, the color intensity of both the T-line and C-line deepened, yet the inhibition rate decreased. Consequently, 6 μL was selected for further studies, as it offered a deeper color and a higher inhibition rate.

#### 3.2.6. TRO-EDC-BSA Concentration

The optimization results for TRO-EDC-BSA concentration are shown in Figure 3e and Figure S1e. The color intensity of the T-line in the negative control varied slightly with changes in the TRO-EDC-BSA concentration. At a TRO-EDC-BSA concentration of 1.8 mg/mL, the inhibition rate for positive samples was the highest. Therefore, 1.8 mg/mL was selected as the concentration for further research.

#### 3.2.7. IgG Concentration

The optimization results for IgG concentration are shown in Figure 3f and Figure S1f. When the IgG concentration was less than 1.0 mg/mL, the color intensity of the C-line on the test strip was relatively weak and did not provide a clear quality control effect. Considering both the inhibition rate and color intensity, an IgG concentration of 1.4 mg/mL was chosen for further studies.

#### 3.2.8. Sample Diluent

The composition of the sample diluent affects the chromatography speed and color development. As shown in Figure 3g and Figure S1g, when a 0.01 M PBS buffer containing 1% Tween 20 was used as the sample diluent, the T-line color intensity of the negative control group was high, and the inhibition rate for positive samples was also high. Therefore, the 0.01 M PBS buffer containing 1% Tween 20 was selected as the sample diluent for honey samples in subsequent studies.

#### 3.2.9. Incubation and Chromatography Time

As depicted in Figure 3h, when the incubation time was 3 min, the T-line color intensity and the inhibition rate of the positive samples in the negative control group attained their maximum values. Hence, an incubation time of 3 min was selected. As shown in Figure 3i, chromatography time had minimal impact on the inhibition rate of positive samples. Meanwhile, the T-line color intensity of the negative control group stabilized at 14 min. Consequently, a chromatography time of 14 min was chosen as the reaction condition for the immunochromatographic test strip.

### 3.3. Analytical Performance

#### 3.3.1. Detection Sensitivity

**Figure 4:**
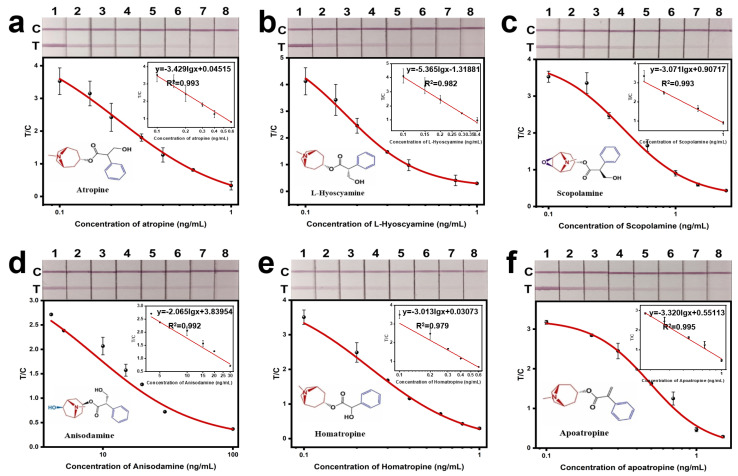
Establishment and linear fitting of standard curves for 6 TAs. (**a**) Atropine. (**b**) L−Hyoscyamine. (**c**) Scopolamine. (**d**) Anisodamine. (**e**) Homatropine. (**f**) Apoatropine.

As shown in Figure 4a–f, when the concentrations of ATR, LHY, SCO, ANI, HOM, and APO were 1 ng/mL, 1 ng/mL, 2.5 ng/mL, 0.1 μg/mL, 1 ng/mL, and 1.5 ng/mL, respectively, the T-line on the test strip disappeared visibly to the naked eye. Since honey samples were diluted five times during detection, the visual detection limits (vLODs) for actual honey samples were 5 ng/mL for ATR, 5 ng/mL for LHY, 12.5 ng/mL for SCO, 0.5 μg/mL for ANI, 5 ng/mL for HOM, and 7.5 ng/mL for APO. Data were read using a test strip reader, and standard curves were established with TA concentration on the x-axis and test strip readings on the y-axis for quantitative analysis. It was observed that the concentrations of the six TAs had good correlations with the color intensity on the test strips.

As shown in Table 1, in the tests of actual honey samples, the linear detection range for ATR was 0.40–3.03 ng/mL, with an IC50 of 1.11 ng/mL and an LOD of 0.22 ng/mL; for LHY, the linear detection range was 0.43–1.70 ng/mL, with an IC50 of 0.86 ng/mL and an LOD of 0.29 ng/mL; for SCO, the linear detection range was 0.83–4.48 ng/mL, with an IC50 of 1.78 ng/mL and an LOD of 0.51 ng/mL; for ANI, the linear detection range was 12.98–150.14 ng/mL, with an IC50 of 44.14 ng/mL and an LOD of 6.34 ng/mL; for HOM, the linear detection range was 0.49–2.87 ng/mL, with an IC50 of 1.19 ng/mL and an LOD of 0.30 ng/mL; for APO, the linear detection range was 1.35–4.51 ng/mL, with an IC50 of 2.46 ng/mL and an LOD of 0.94 ng/mL. Using ATR as the reference, the cross-reactivity rates for LHY, SCO, ANI, HOM, and APO were calculated to be 129.1%, 62.2%, 2.5%, 93.1%, and 45.0%, respectively.

These results indicate that the AuNPs-LFIA method can achieve a semi-quantitative and quantitative analysis of the six TAs with excellent detection sensitivity.

#### 3.3.2. Specificity

**Figure 5:**
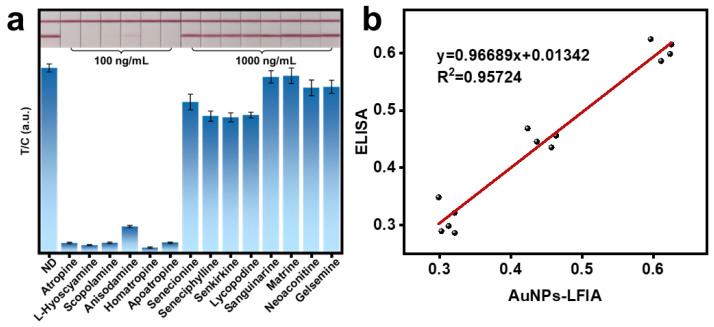
Evaluation of method specificity and accuracy. (**a**) Evaluation of the specificity of the AuNPs-LFIA method. (**b**) Comparison with the ELISA method.

The specificity of the established AuNPs-LFIA method was evaluated by analyzing fourteen different alkaloids and comparing their T/C values. As shown in Figure 5a and Figure S3, significant inhibition was observed only for the six target TAs, indicating that the AuNPs-LFIA method possesses good detection specificity. Using atropine as the target analyte, the detection results of the AuNPs-LFIA method were consistent with those of the ELISA method, indicating that the AuNPs-LFIA method is accurate and reliable (Figure 5b).

#### 3.3.3. Accuracy and Reliability

To evaluate the accuracy and reliability of the established LFIA method, spiked recovery tests were conducted for the six TAs in honey samples. The honey samples were spiked with TAs at low, medium, and high concentrations and then tested using the test strips. As shown in Table 2, the recovery rates for the six TAs ranged from 90.1% to 109.4%, with coefficients of variation (CV) less than 14.7%. These results indicate that the method possesses good accuracy and precision.

#### 3.3.4. Practicality

As shown in Figure S4, using three kinds of honey diluted five times as the matrix for the spiked detection of atropine, there is no significant difference in the detection results, confirming the robustness of the AuNPs-LFIA method. As shown in Figure S5, using six common commercial plant-based beverages as the matrix (adding 1% Tween 20) for the spiked detection of atropine, in all detection groups, the T-line can be completely eliminated when the atropine content is 1 ng/mL, confirming the practicability of the AuNPs-LFIA method.

## 4. Conclusions

As a very common food eaten by a huge consumer population, honey requires stricter supervision in terms of quality and safety. Therefore, there is an urgent need to develop more convenient and rapid detection methods to meet the need for on-site rapid detection. In this study, for the first time, an AuNPs-LFIA method that can be used for rapid detection of six possible TAs in honey was established, filling the gap in the detection of tropane alkaloids in honey by using the LFIA method. By optimizing a series of parameters that could affect the performance of the AuNPs-LFIA method, it was determined that the method exhibits excellent sensitivity for the six TAs in honey, enabling semi-quantitative and quantitative analysis within 17 min. Specificity tests and spiked recovery experiments demonstrated that the method possesses good specificity, accuracy, and reliability. The AuNPs-LFIA method developed in this study can be used for the on-site rapid detection of 6 tropane alkaloids in honey. However, in this study, the acquisition of quantitative data still relied on a grayscale instrument. In the future, it is hoped that it can be combined with portable signal reading devices such as smartphones to achieve quantitative analysis of detection targets more quickly.

## Figures and Tables

**Table 1:** LOD, linear range, and cross-reactivity of TAs detected by test strips.

Analytes	R^2^	IC_50_ (ng/mL)	LOD (ng/mL)	Linear Range (ng/mL)	Cross-Reactivity
ATR	0.998	1.11	0.22	0.40–3.03	100%
LHY	0.999	0.86	0.29	0.43–1.70	129.1%
SCO	0.998	1.78	0.51	0.83–4.48	62.2%
ANI	0.996	44.14	6.34	12.98–150.14	2.5%
HOM	0.997	1.19	0.30	0.49–2.87	93.1%
APO	0.998	2.46	0.94	1.35–4.51	45.0%

**Table 2:** Recovery and coefficient of variation for the analysis of honey (n = 3).

Analytes	Spiked Concentration (ng/mL)	Tested Concentration (ng/mL)	Mean Recovery (%)	Coefficient of Variation (%)
ATR	0.5	0.55 ± 0.01	109.4	1.4
	1.0	0.93 ± 0.08	93.4	8.5
	3.0	3.15 ± 0.03	105.3	1.0
LHY	0.5	0.48 ± 0.02	95.6	3.8
	1	1.01 ± 0.10	100.8	9.9
	1.5	1.54 ± 0.05	102.8	3.5
SCO	1.0	1.03 ± 0.09	103.2	8.6
	2.0	2.10 ± 0.19	104.8	9.1
	4.0	3.76 ± 0.15	93.9	3.9
ANI	15	14.72 ± 1.66	98.1	11.2
	45	45.74 ± 3.46	101.6	7.6
	150	140.26 ± 7.54	93.5	5.4
HOM	0.5	0.53 ± 0.08	106.7	14.7
	1	0.90 ± 0.03	90.1	2.9
	2.5	2.63 ± 0.25	105.1	9.3
APO	1.5	1.56 ± 0.10	103.9	6.3
	2.5	2.63 ± 0.17	105.2	6.4
	4.5	4.53 ± 0.14	100.7	3.1

## Data Availability

No new data were created or analyzed in this study.

## Supplementary Materials

The following supporting information can be downloaded at: https://www.mdpi.com/article/10.3390/s24227265/s1, Figure S1: Photos of condition optimization, Figure S2: The standard curve and linear fitting of the ELISA method, Figure S3: Evaluation of the specificity of the AuNPs-LFIA method (Supplementary experiment), Figure S4: Evaluation of interference of different types of honey on detection results, Figure S5: Evaluation of interference of different commercial beverages on detection results.
