# Supplementary material for: Strategy for Accurate Detection of Six Tropane Alkaloids in Honey Using Lateral Flow Immunosensors
Source: Sensors (Basel). 2024 Nov 13;24(22):7265. doi: 10.3390/s24227265 (PMC11598261; doi:10.3390/s24227265)
Supplement: Supplementary file 1 [file sensors-24-07265-s001.zip › sensors-3220711-supplementary.pdf]

Supplementary Information

Strategy for Accurate Detection of Six Tropane Alkaloids in Honey Using Lateral Flow Immunosensors

Bo-yan Sun[1], Chuan-lei Wang[1], Zi-le Wang[1,3], Jia-yi Liang[2], Ke Han[1], Shuai Zhang[1], Chun-chao Yin[1], Xiao-mei Wang[1], Chu-jun Liu[1], Zhi-yue Feng[1], Si-han Wang[2*] and Hai-yang Jiang[1*]

1 Department of Veterinary Pharmacology and Toxicology, National Key Laboratory of Veterinary Public Health Security, Beijing Key Laboratory of Detection Technology for Animal-Derived Food Safety, Beijing Laboratory for Food Quality and Safety, College of Veterinary Medicine, China Agricultural University, Beijing 100193, People's Republic of China

2 Department of Chemistry, Waterloo Institute for Nanotechnology, University of Waterloo, Waterloo, Ontario N2L 3G1, Canada

3 Chinese Academy of Inspection and Quarantine, Beijing 100176, People's Republic of China

* Corresponding Author:

Sihan Wang

Email: s2298wan@uwaterloo.ca

Haiyang Jiang

Email: haiyang@cau.edu.cn

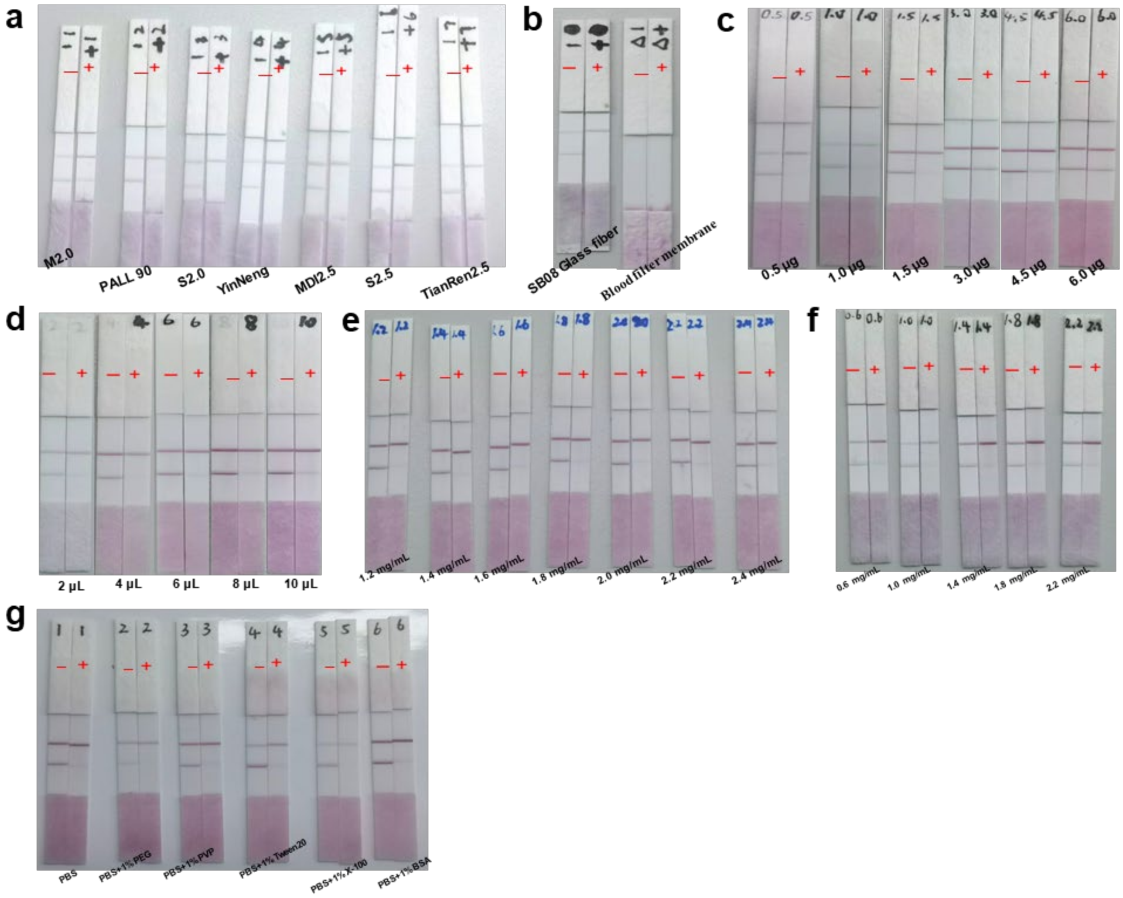

Figure S1. Photos of condition optimization. **(a)** Optimization of NC membrane type. **(b)** Optimization of sample pad types. **(c)** Optimization of TAs antibody input amount. **(d)** Optimization of AuNPs-Abs input amount. **(e)** Optimization of TRO-EDC-BSA concentration. **(f)** Optimization of IgG concentration. **(g)** Optimization of sample diluent.

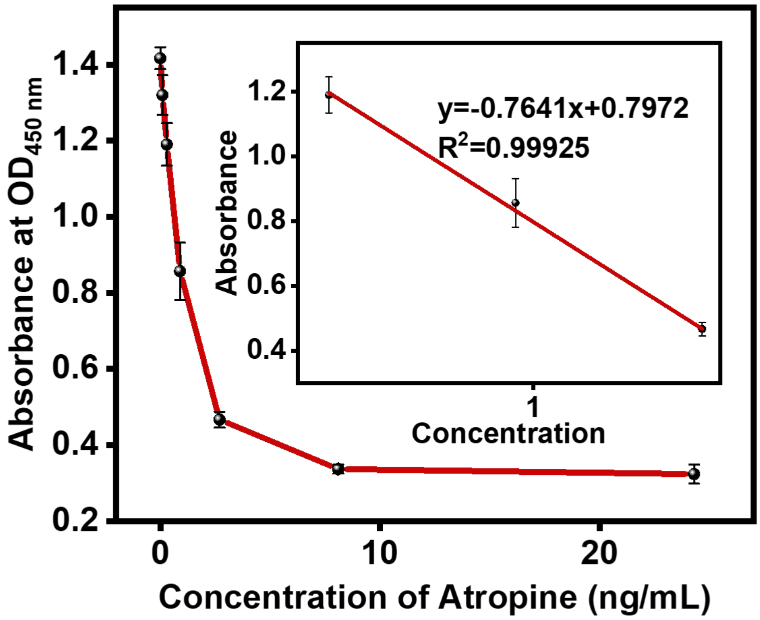

Figure S2. The standard curve and linear fitting of the ELISA method.

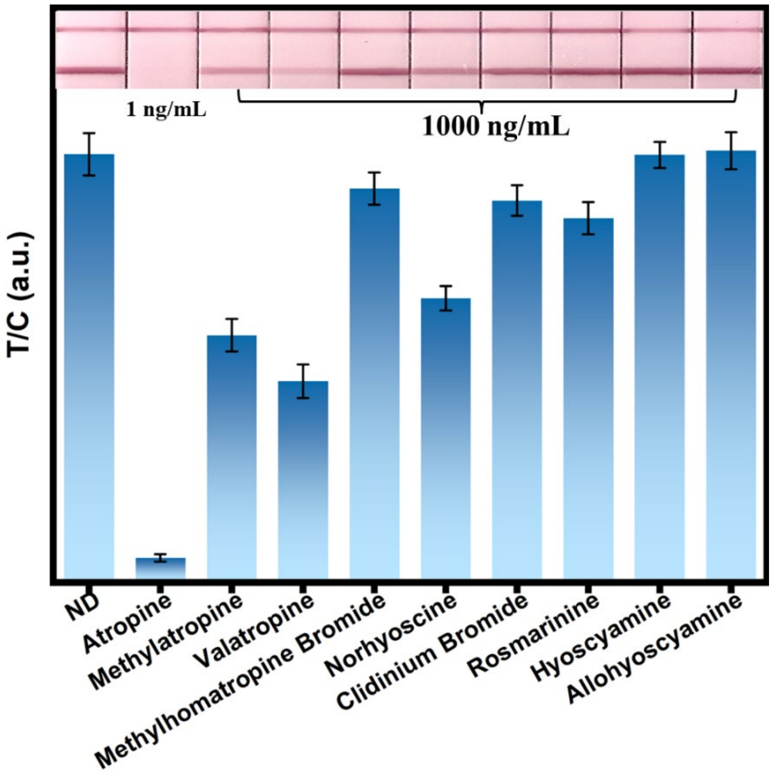

Figure S3. Evaluation of the specificity of the AuNPs-LFIA method (Supplementary experiment).

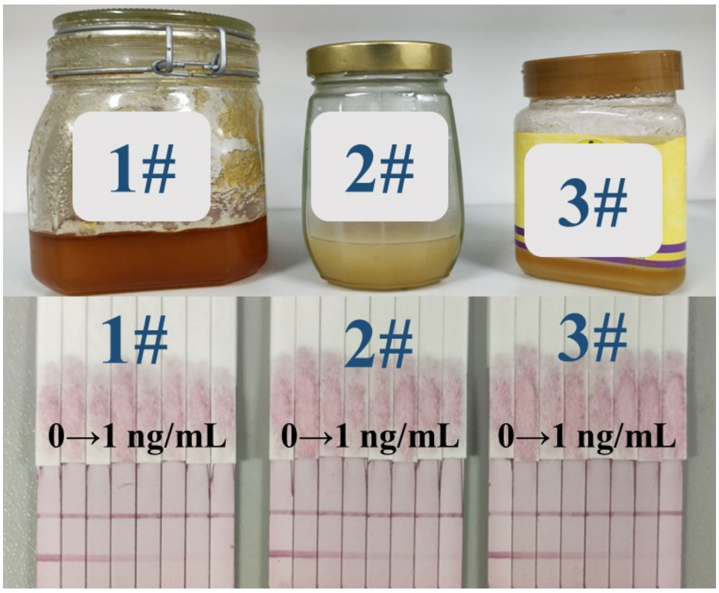

Figure S4. Evaluation of interference of different types of honey on detection results.

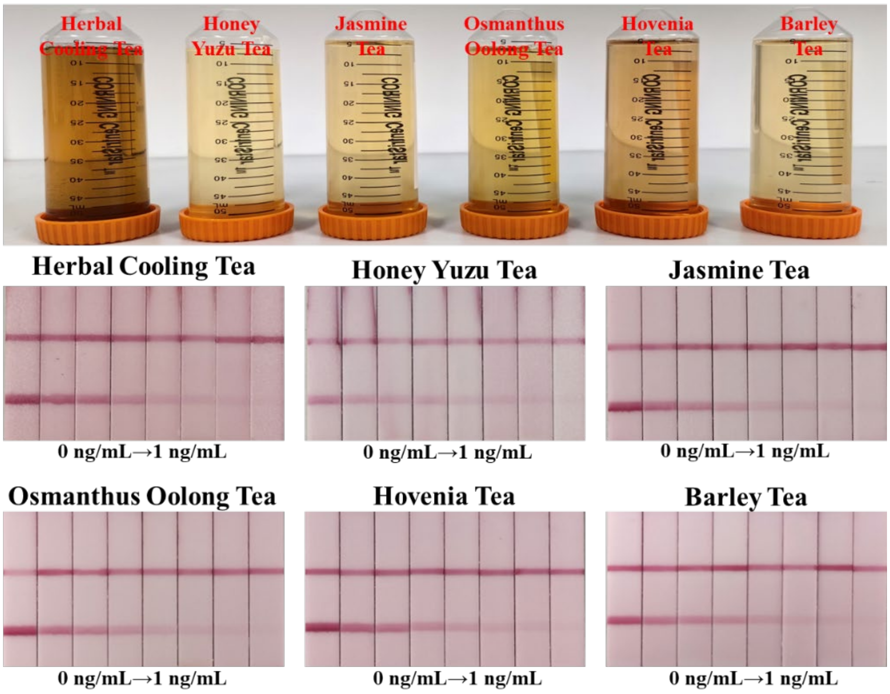

Figure S5. Evaluation of interference of different commercial beverages on detection results.
